# The first colorimetric receptor for the B_4_O_7_^2−^ anion based on nitro substituted phenanthroimidazole ferrocene derivatives†

**DOI:** 10.1039/c7ra12700f

**Published:** 2018-01-19

**Authors:** Pei Wu, Guo Wang, Lu Zhou, Jing Lu, Jianchun Wang

**Affiliations:** Department of Chemistry, Capital Normal University Beijing 100048 P. R. China cnuwjc@cnu.edu.cn

## Abstract

Four phenanthroimidazole ferrocene derivatives (2a–2d) were designed, synthesized and characterized by ^1^H NMR, ^13^C NMR and high-resolution mass spectroscopy (HRMS). Recognition of 12 anions by 2a–2d was investigated by UV-Vis absorption analysis, showing that 2b and 2d sensed B_4_O_7_^2−^ selectively among the tested anions with an obvious color change observed. ^1^H NMR titrations and theoretical calculations demonstrated that 2b binds B_4_O_7_^2−^ through O⋯H hydrogen bonding and O⋯B interactions between the nitro moiety and the B_4_O_7_^2−^ anion.

## Abstract 2

Four phenanthroimidazole ferrocene derivatives (2a–2d) were synthesized. Recognition of 12 anions by 2a–2d was investigated by UV-Vis absorption analysis, showing that 2b and 2d sensed B_4_O_7_^2−^ selectively with an obvious color change observed.

## Graphical abstract

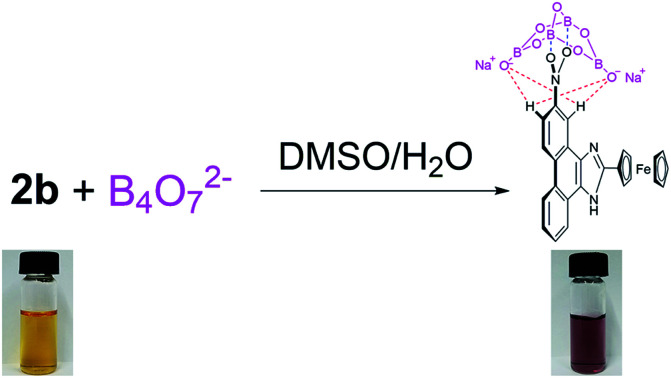

## 1. Introduction

Sodium borate (Na_2_B_4_O_7_·10H_2_O) is an important boron-containing mineral and is used widely in the fields of glassmaking, metallurgy, detergents, cosmetics, pesticides and medicines. The borate anion, or tetraborate anion, is a bicyclic structure that is composed of two BO_4_ and two BO_3_ moieties. A receptor for sensing the borate anion is urgently needed because about five hundred thousand tons of sodium borate is produced in China every year. The massive industrial production leads to the production of a large amount of waste water containing borate anions, and no receptor to detect this anion exists.

Imidazole is a five-membered heterocyclic aryl system that coordinates easily with metal ions to form imidazole complexes. Different structural ferrocenyl imidazole derivatives can coordinate with cations, such as Ag(i), Cu(i),^1^ Pd(ii), Pt(ii),^2^ Mo(ii),^3^ Mn(ii), Co(ii),^4^ Pb(ii)^5^ and Hg(ii),^6^ to form complexes, and can be used in the recognition of cations. Imidazole can also interact with anions by hydrogen bonding, electrostatic forces and other non-covalent interactions to function as a complexing unit of an anion sensor. Ferrocene is a good sensing unit because of its excellent electrochemical properties. In 2002, Tomas and coworkers reported a simple ferrocenyl imidazolium, in which ferrocene bonded to the nitrogen of imidazole by a methylene group could interact with Cl^−^, Br^−^, NO_3_^−^ and HSO_4_^−^ anions,^7^ but with broad selectivity. In recent years, a series of acyclic and trinuclear ferrocene-based imidazolium receptors noticeably improved selectivity toward the F^−^ anion.^8–10^ Structural characterization of other ferrocenyl imidazole derivatives showed that specific recognition properties involve the ferrocene being linked directly with the C2 of imidazole, with the C4 and C5 fused to a conjugative system. One of the receptors discovered was 2-ferrocenyl-1*H*-anthra[1,2-*d*]imidazole-6,11(5*aH*,11*aH*)-dione, which was capable of detecting [CN^−^]_aq_ as low as ∼0.1 ppm.^11^ The ferrocene–imidazopyrene dyad is a simple but effective dual redox and fluorescent ion pair receptor that can detect Hg^2+^ and H_2_PO_4_^−^ ions simultaneously.^12^ Another ion pair sensor is the bisferrocene–benzobisimidazole triad, which acts as a multichannel receptor for selective sensing of Hg^2+^ and HSO_4_^−^ ions.^13^

We are interested in studying the synthesis of ferrocenyl imidazole sensors^14^ and the application of these sensors as cation and anion receptors.^15,16^ Ferrocene moiety in these receptors raised the selectivity. It was due to the electronic properties and rigid structure of ferrocene. 2-Ferrocenyl-1*H*-phenanthro-[9,10-*d*]imidazole (2a) is an electrochemical switching device.^17^ When studying the UV-Vis spectral response of 2a to different ions, we found that the absorbance at 400 nm in the presence of borate and hydroxyl anions showed significant increases, but no color change to the solution was observed. In this report, a strategy was designed to introduce a nitro group to ferrocenyl imidazophenanthrene and link an acetyl group to the cyclopentadienyl (Cp) to yield novel receptors (2b–2d) that display better sensing responses to the borate anion.

## 2. Results and discussion

### 2.1 Synthesis of receptors

**Scheme 1:**
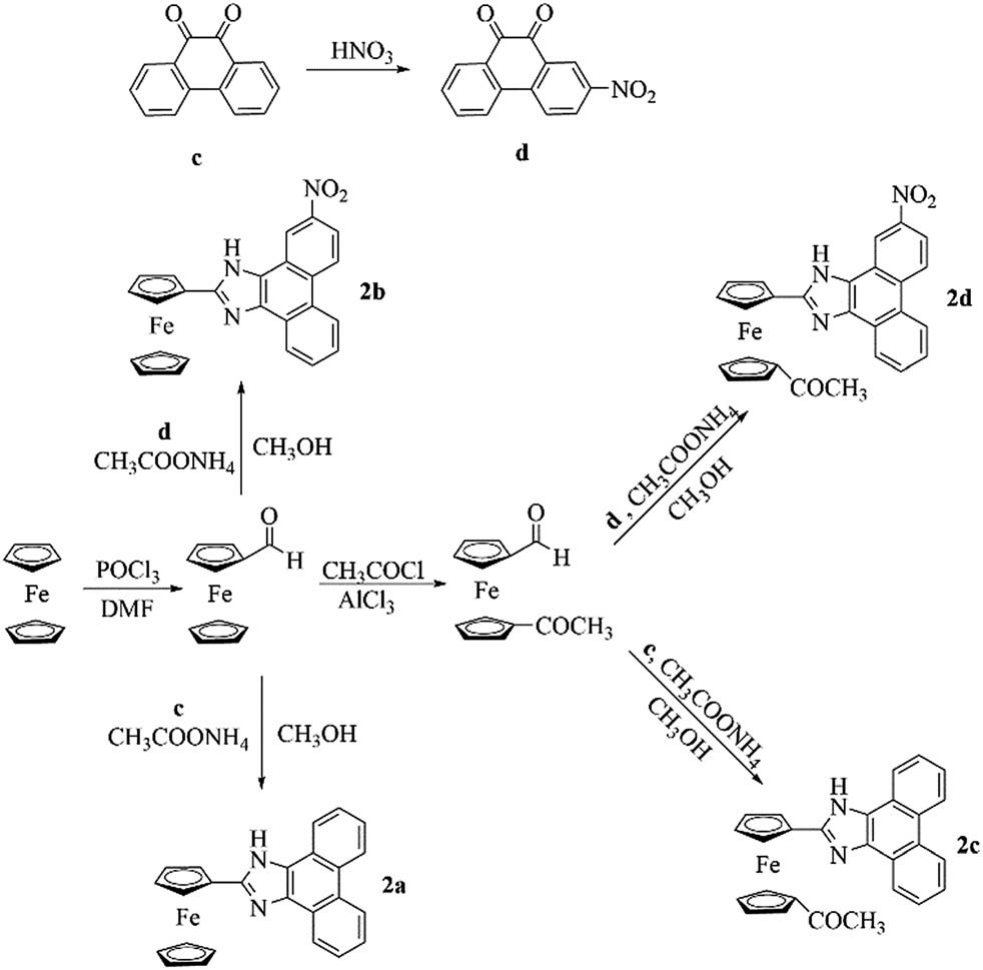
Synthesis of 2a–2d.

Methods for the syntheses of the receptors (2a–2d) are shown in Scheme 1. 2-Nitrophenanthraquinone (d) was prepared by the nitration of phenanthraquinone (c).^18^ In the presence of NH_4_OAc, formylferrocene and 1′-acetyl-1-formylferrocene reacted with c and d, respectively, and underwent Radziszewski synthesis to yield the products (2a–2d).

### 2.2 X-ray diffraction single crystal structure of 2a

**Fig. 1:**
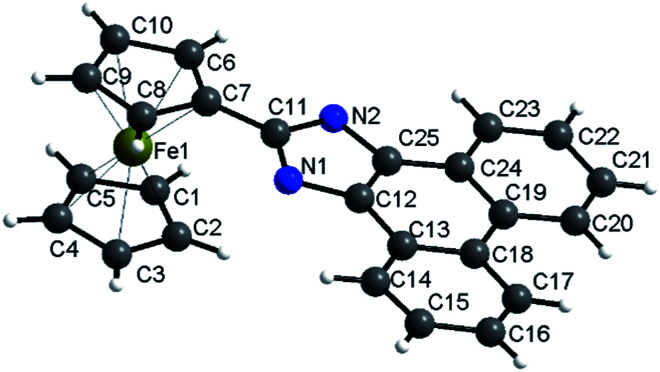
Molecular structure of 2a. CCDC: 1509214.†

The X-ray diffraction single crystal structure of 2a is shown in Fig. 1. The Fe–C bond length ranges between 2.043 and 2.059 Å. The dihedral angle of the two Cps is 0.217°, and the dihedral angle between Cp and phenanthroimidazole is 5.055°. The structure shows that Cp and phenanthroimidazole are almost parallel and form a larger conjugative system.

The rigid structure of ferrocene and phenanthroimidazole provides a specific space configuration to facilitate selectivity toward anions.

### 2.3 UV-Vis spectrum

**Fig. 2:**
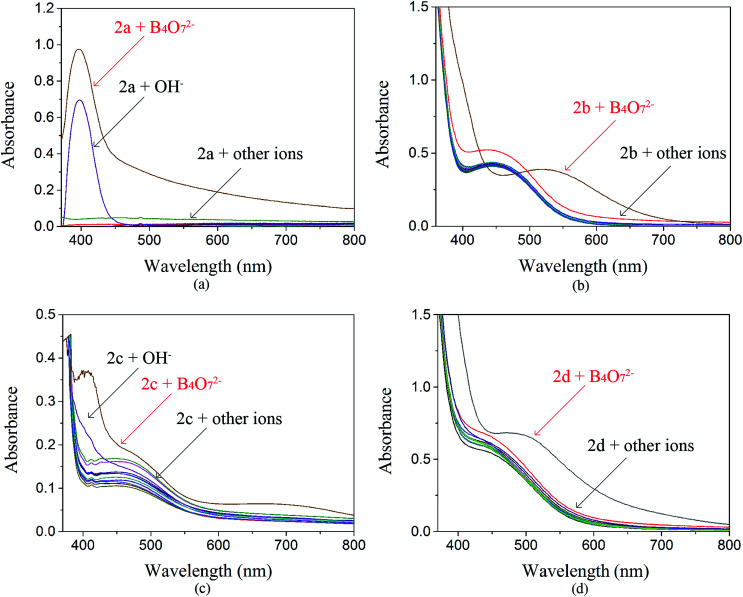
Influence of various anions on the UV-Vis spectra of 2a–2d.

**Fig. 3:**
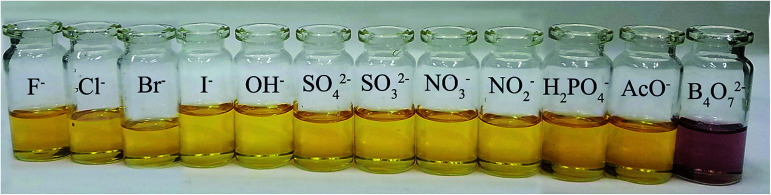
Influence of various anions on colorimetric properties of 2b.

**Fig. 4:**
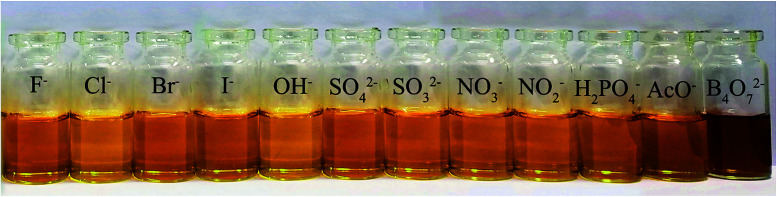
Influence of various anions on the colorimetric properties of 2d.

**Fig. 5:**
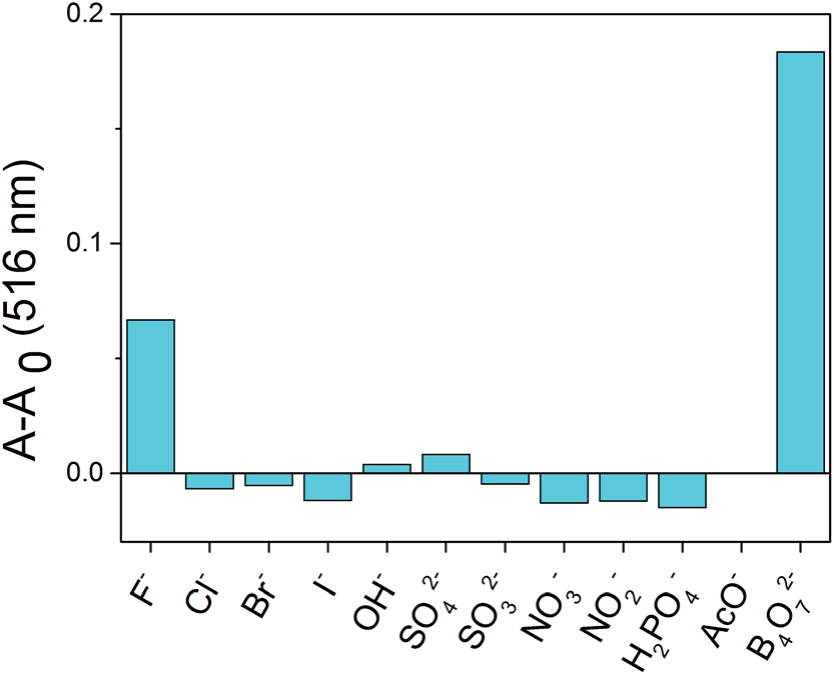
Relative absorbance at 516 nm of different anions in the presence of 2b.

The anion recognition properties of the receptors 2a–2d toward F^−^, Cl^−^, Br^−^, I^−^, OH^−^, SO_4_^2−^, SO_3_^2−^, NO_3_^−^, NO_2_^−^, H_2_PO_4_^−^, AcO^−^ and B_4_O_7_^2−^ were evaluated by UV-Vis spectroscopy. The solvent used in ion recognition manipulation was a mixture of DMSO and water (*V*_DMSO_ : *V*_H_2_O_ = 4 : 1). The solutions of 2a–2d were prepared in DMSO (*c* = 1.0 × 10^−3^ mol L^−1^). All the tested anions were dissolved in water (*c* = 1.0 × 10^−2^ mol L^−1^). The receptor 2a was studied by UV-Vis spectroscopy. The absorbance at 400 nm increased dramatically when B_4_O_7_^2−^ and OH^−^ were added to 2a (Fig. 2(a)), but both anions showed the same yellow color. The receptor 2c possesses one acetyl group more than 2a. When the interaction of 2c with 12 anions was investigated, we observed that the maximum absorbance in the UV-Vis spectrum of 2c in the presence of B_4_O_7_^2−^ was at 400 nm, whereas the absorbance maximum of the peak for 2c in the presence of OH^−^ had blue shifted (Fig. 2(c)). No color change was observed. Addition of B_4_O_7_^2−^ to 2b, which has one more nitro group than 2a, caused an immediate change in the color of the solution from light yellow to violet (Fig. 3). The change in the spectrum was caused by a bathochromic shift in the absorption maxima from 436 to 516 nm (Fig. 2(b)) and a change in the molar extinction coefficient from 4265 to 3890 L (mol cm^−1^)^−1^, when 10 equivalents in concentration of B_4_O_7_^2−^ was added. As shown in Fig. 5, 2b shows higher selectivity toward B_4_O_7_^2−^ over other anions. The receptor 2d possesses one more acetyl group and one more nitro group than 2a. Addition of B_4_O_7_^2−^ to a solution of 2d caused an immediate color change from yellow to brown (Fig. 4). Correspondingly, the absorption maxima shifted from 436 to 496 nm (Fig. 2(d)) and the molar extinction coefficient from 5628 to 6694 L (mol cm^−1^)^−1^, when 10 equiv. of B_4_O_7_^2−^ was added.

For the tested anions, receptors 2b and 2d possessed sensing properties only toward B_4_O_7_^2−^. Thus, introduction of a nitro group to phenanthroimidazole resulted in a prominent color change, whereas the acetyl group showed no effect.

### 2.4 Competition with other anions

**Fig. 6:**
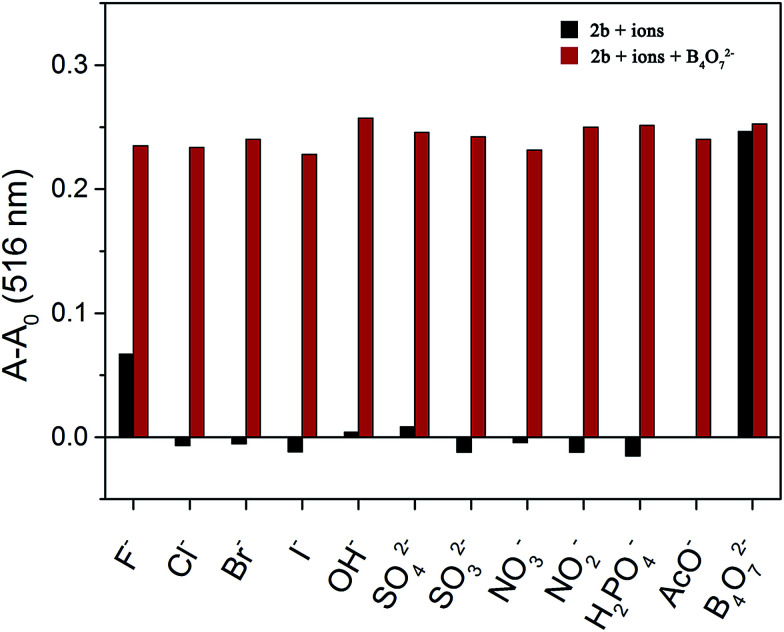
Competitive selection of B_4_O_7_^2−^ by 2b in the presence of other anions (10 equiv. in DMSO/H_2_O (4 : 1)).

A competition experiment was performed to test the specificity of 2b toward B_4_O_7_^2−^. No significant interference was observed in the presence of competing anions and the absorbance enhancement induced by B_4_O_7_^2−^ was retained (Fig. 6). Interestingly, all the anions were unable to decrease the absorbance of the 2b–B_4_O_7_^2−^ complex. Therefore, none of the anions test could alter the affinity of 2b towards B_4_O_7_^2−^.

### 2.5 UV-Vis spectral response of 2b toward a concentration gradient of B_4_O_7_^2−^

**Fig. 7:**
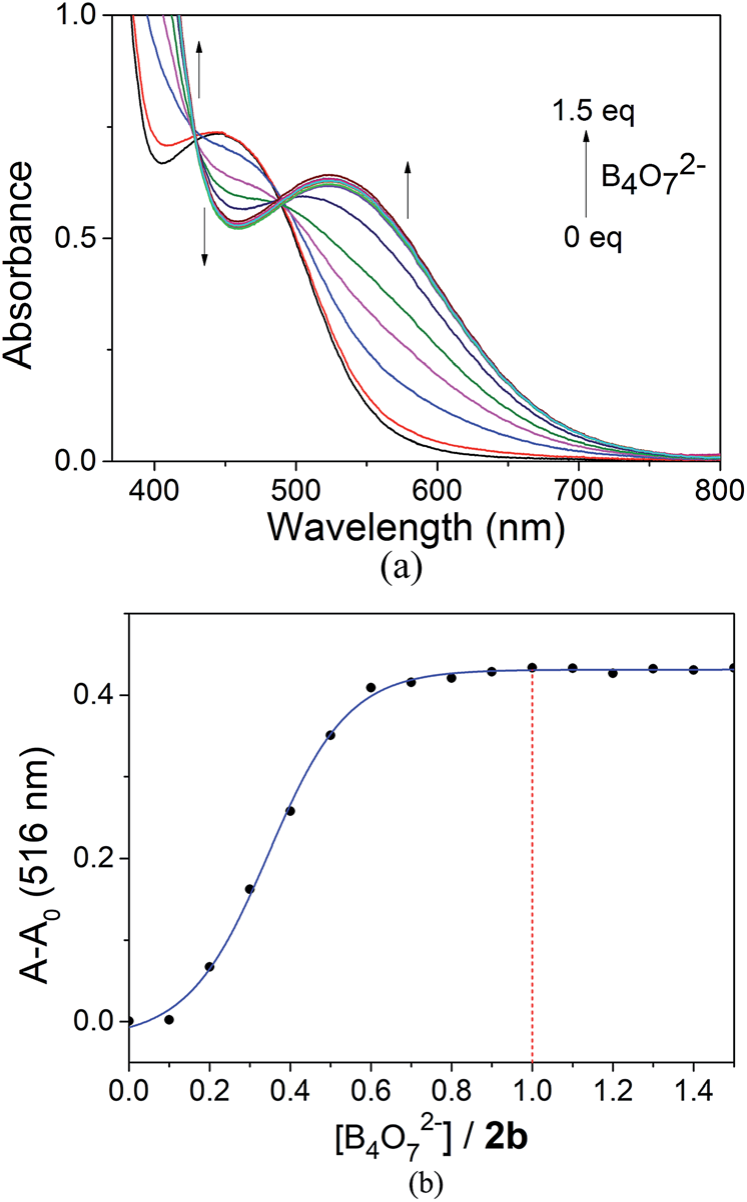
(a) Absorbance spectra of 2b with B_4_O_7_^2−^. (b) Relative absorbance at 516 nm of 2b in the presence of B_4_O_7_^2−^ at different B_4_O_7_^2−^ : 2b ratios.

The 2b to B_4_O_7_^2−^ anion complex ratio was determined. At 20 °C, titrations were carried out in a DMSO/H_2_O (4 : 1) solution and the absorbance was monitored. The experiments were performed by preparing a solution (3 × 10^−4^ mol L^−1^) of 2b in DMSO/H_2_O (4 : 1), followed by the addition of aqueous sodium borate (Fig. 7(a)). No obvious change in color was observed when the concentration of B_4_O_7_^2−^ was below 1 : 2. At molar ratios of 2b and the B_4_O_7_^2−^ anion greater than 1 : 2, a color change to lavender was observed and the *λ*_max_ showed a slight red shift. The color changed to violet when the concentration of B_4_O_7_^2−^ reached 1 equiv. to 2b. At higher concentrations of B_4_O_7_^2−^ no further change in the color was observed and the UV-Vis spectrum did not change either (Fig. 7(b)). This observation showed that the stoichiometry of the receptor 2b to B_4_O_7_^2−^ anion complex was 1 : 1.

### 2.6 Measurement of Job's plot, LOD and *K*_a_ of 2b toward the B_4_O_7_^2−^ anion

**Fig. 8:**
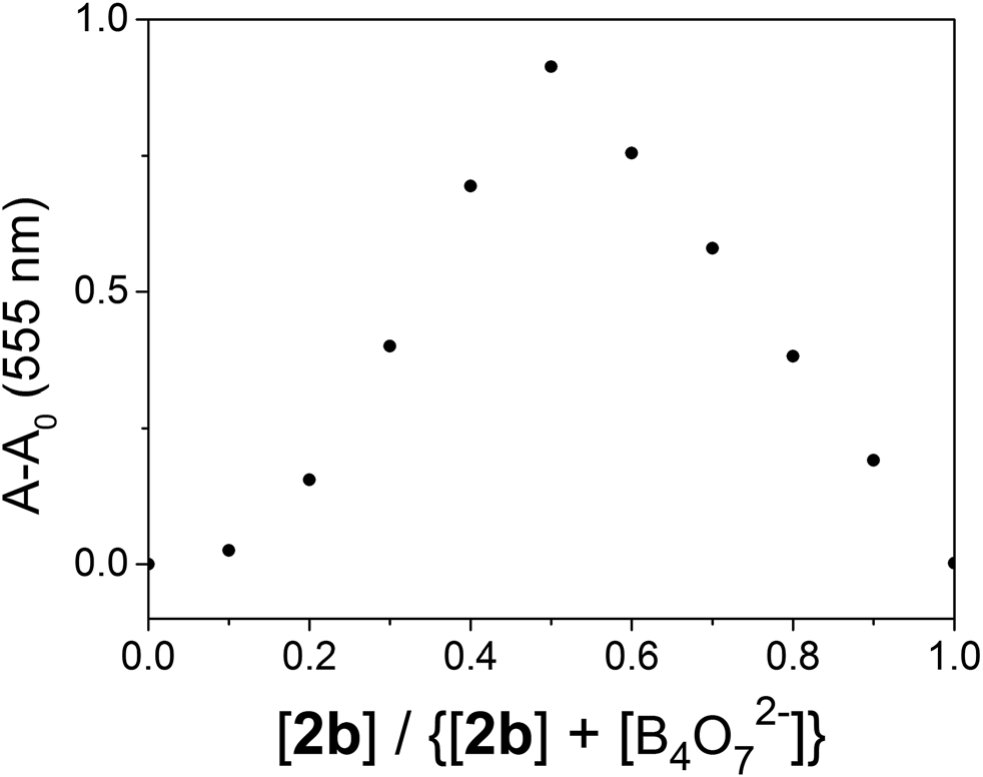
Job's plot of the interaction of 2b with B_4_O_7_^2−^ in DMSO/H_2_O (4 : 1).

**Fig. 9:**
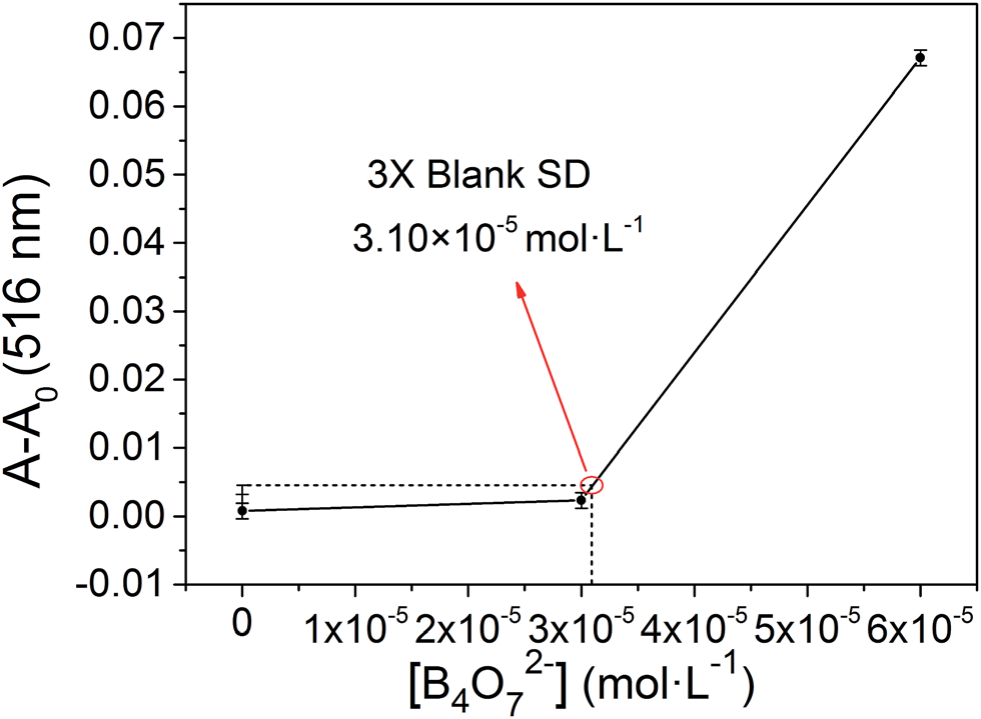
The absorbance of various B_4_O_7_^2−^ concentrations measured with respect to the absorbance of the blank.

The binding stoichiometry of 2b with B_4_O_7_^2−^ was examined by the Job's plot method. The presence of a maximum absorbance at a mole fraction of 0.5 shows the formation of 1 molecule of 2b with 1 molecule of B_4_O_7_^2−^. The binding mode is presented in Fig. 8, with a limit of detection (LOD) of 3.10 × 10^−5^ mol L^−1^, as estimated by the 3*σ* rule (Fig. 9). The binding constant of 2b with B_4_O_7_^2−^ is 5.45 × 10^4^, which was obtained from the absorbance titration data, using the Benesi–Hildebrand equation (Fig. S1†):
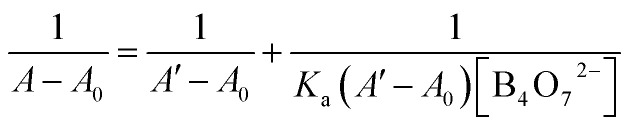
where *A*_0_ is the absorbance of 2b without B_4_O_7_^2−^, *A* is the absorbance with a particular concentration of B_4_O_7_^2−^, *A*′ is the absorbance of the fully complexed form at the highest concentration of B_4_O_7_^2−^, and *K*_a_ is the binding constant.

### 2.7 Mechanism study

**Fig. 10:**
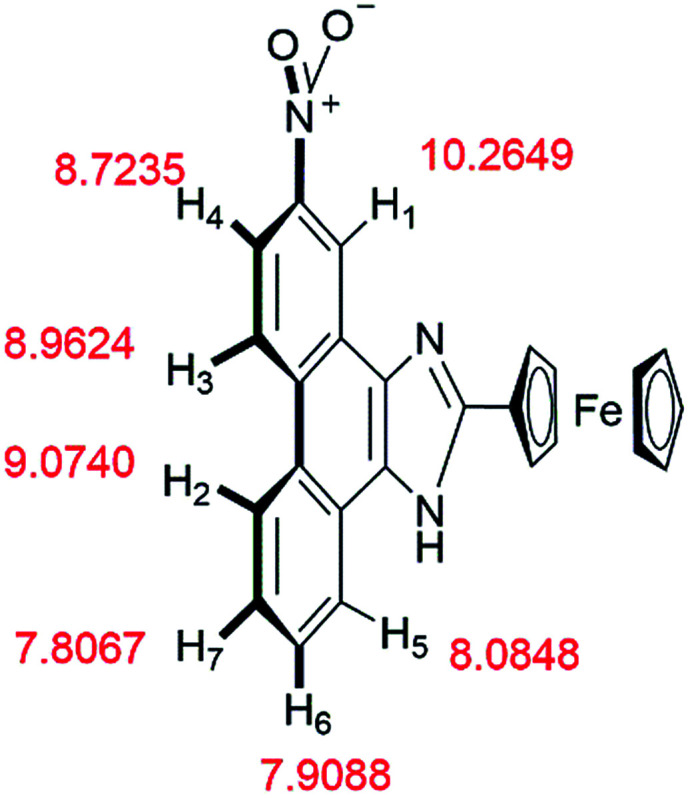
Chemical shifts of compound 2b calculated by Gaussian 03.

The mechanism of 2b binding to B_4_O_7_^2−^ was determined by conducting ^1^H NMR titration experiments and theoretical calculations. The chemical shifts of 2b were calculated based on the optimized geometry. The calculations were performed with the hybrid density functional B3LYP^19,20^ and the 6-311++G** basis set using Gaussian 03 ^21^ (Fig. 10). Hydrogen atoms (H_1_–H_7_) on the phenanthrene are labeled with chemical shifts. H_1_ and H_4_ stand for the first and the fourth biggest chemical shift values, correspondingly.

**Fig. 11:**
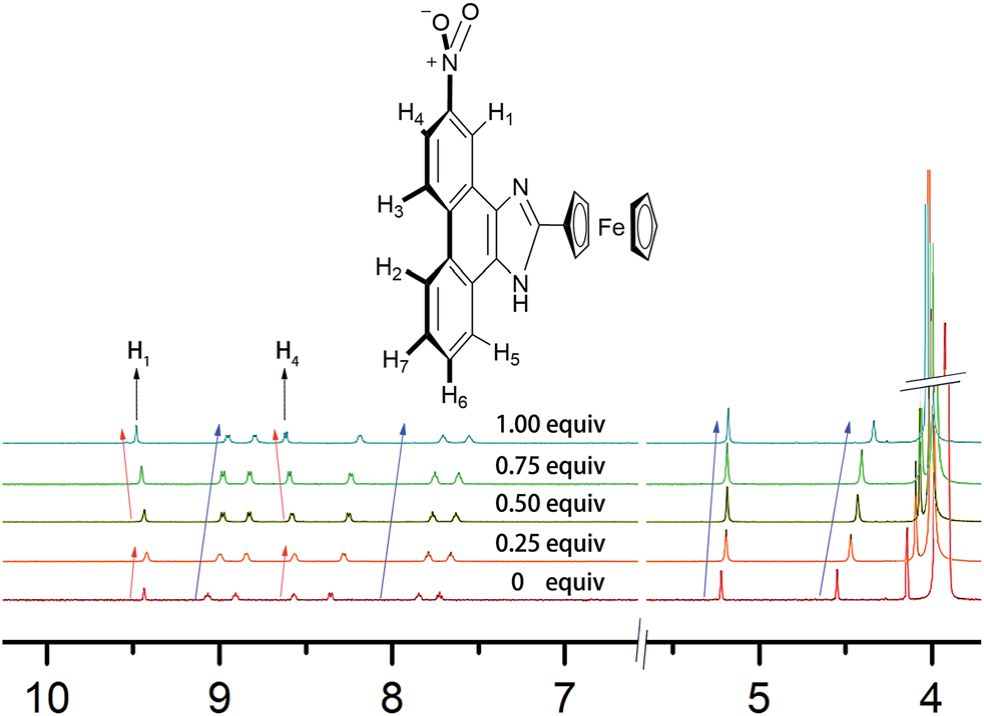
^1^H NMR titration spectra of 2b in DMSO-*d*_6_ in the presence of various equivalents B_4_O_7_^2−^ in D_2_O.

According to the ^1^H NMR titration experiments (Fig. 11), the chemical shifts of H_1_–H_7_ are in the range of 7.4–9.4 ppm. The chemical shifts of most peaks in the NMR spectrum moved upfield as the equiv. of B_4_O_7_^2−^ increased. Only the peaks of H_1_ and H_4_ shifted upfield at equiv. less than 0.25 and then downfield in response to further increases in equiv. of B_4_O_7_^2−^.

**Scheme 2:**
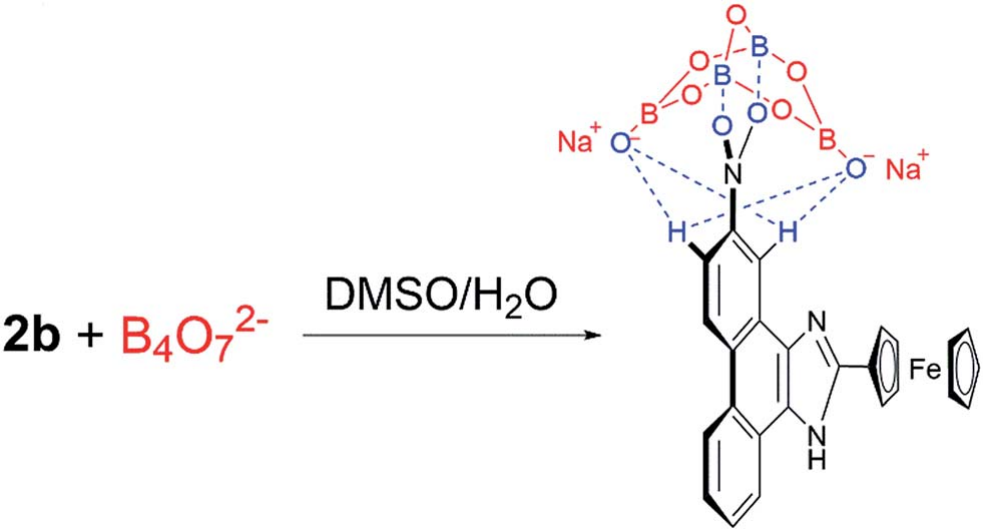
Proposed mechanism describing how 2b detects borate ions.

Based on the ^1^H NMR titration and the calculation results, the following mechanism is proposed (Scheme 2). The static force of borate anion toward 2b and hydrogen bonds between H_1_/H_4_ and two oxygen atoms of B_4_O_7_^2−^ are converse interaction. Two boron atoms of B_4_O_7_^2−^, as an electron-deficient element bind readily with two oxygen atoms of the nitro. This event leads to all peaks in the spectra of 2b shifting upfield initially. The peaks of H_1_ (9.4 ppm) and H_4_ (8.6 ppm) shift downfield at higher B_4_O_7_^2−^ equiv. because of the formation of hydrogen bonds between H_1_/H_4_ and two oxygen atoms of B_4_O_7_^2−^, which support the results of the theoretical calculations.

## 3. Detection of B_4_O_7_^2−^ in lake water

**Fig. 12:**
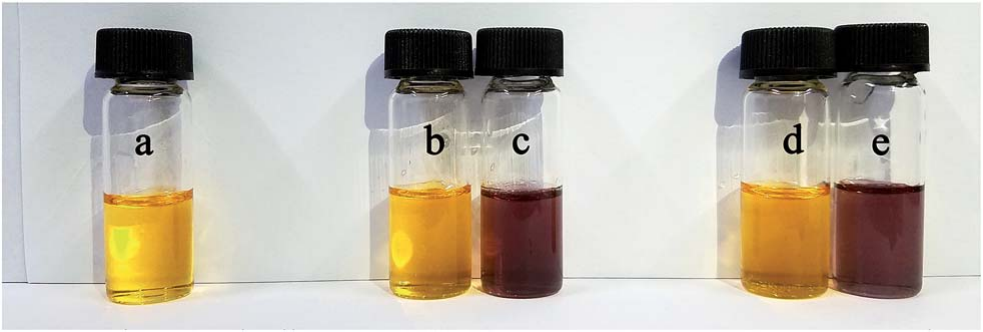
Influence of different water samples on colorimetric properties of 2b (a) 2b; (b) 2b and sample1; (c) 2b, sample 1 and B_4_O_7_^2−^; (d) 2b and sample 2; (e) 2b, sample 2 and B_4_O_7_^2−^. The solvents in bottle a was the mixture of DMSO and deionized water (*V*_DMSO_ : *V*_H_2_O_ = 4 : 1). The solvents in bottle b–e were the mixture of DMSO and lake water (*V*_DMSO_ : *V*_H_2_O_ = 4 : 1).

**Fig. 13:**
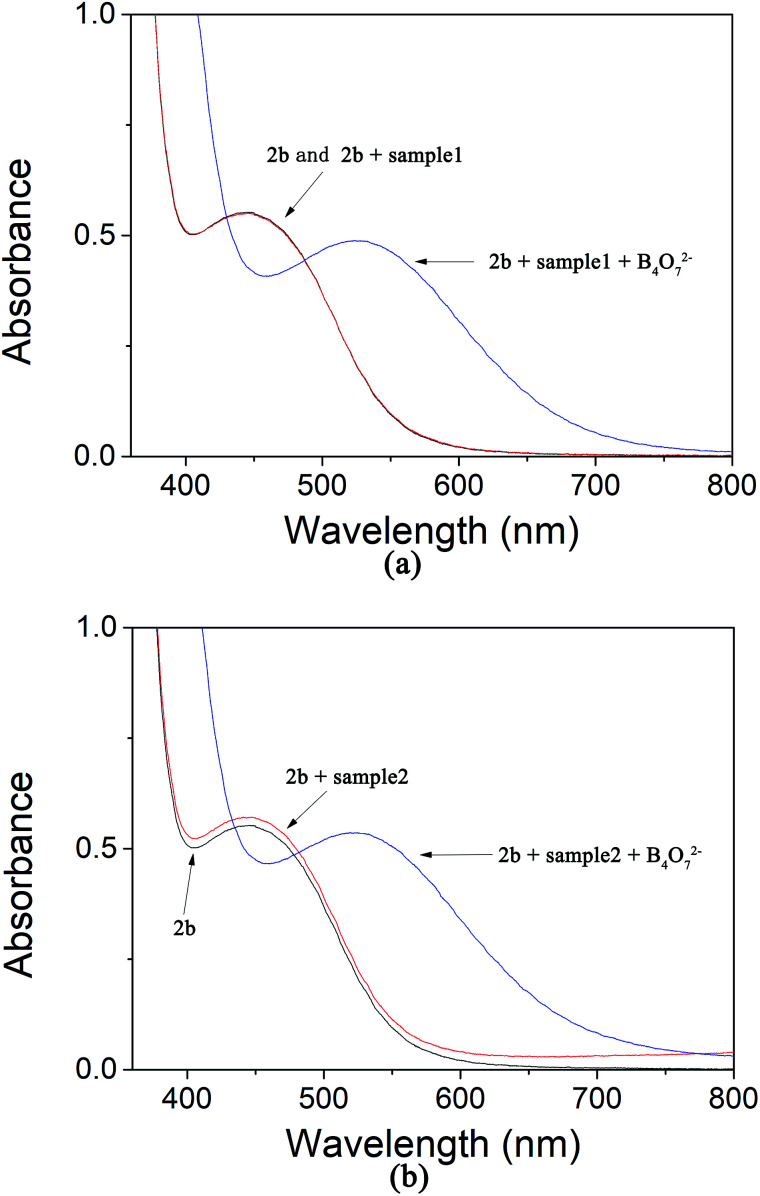
(a) The absorption of 2b in the water samples 1; (b) the absorption of 2b in the water samples 2.

To evaluate whether our colorimetric probe can detect B_4_O_7_^2−^ anion in actual samples, we found some samples in the lakes of Yuanming Yuan (the Old Summer Palace) in Beijing. The insoluble substances were removed by filtration. Then 2b was added in. We observed that sample 1 and 2 showed the same yellow color (Fig. 12(b) and (d)). UV-Vis spectroscopy was used to study the two samples and it showed that no obvious change was found (Fig. 13). It manifested that concentration of B_4_O_7_^2−^ anion in sample 1 and 2 was below the limit of detection. When B_4_O_7_^2−^ anion was added into sample 1 and 2, the color changed from yellow to violet immediately and the change in the spectrum was caused by a bathochromic shift in the absorption maxima from 436 to 516 nm (Fig. 12(c, e) and 13(a, b) the blue curves). It means that the receptor could be used to determine the B_4_O_7_^2−^ anion in actual samples.

## 4. Conclusions

The syntheses of new ferrocenyl phenanthroimidazole derivatives 2a–2d have been achieved using simple and short synthetic routines from ferrocene. The introduction of a nitro group to receptor 2a to afford 2d gave rise to a prominent color change, and 2b and 2d formed 1 : 1 complexes with the borate anion. Both 2b and 2d showed good selectivity toward the borate anion when testing the 12 anions, and selectivity was visibly detected by a color change of the reaction solution.

## 5. Experimental section

### 5.1 Instruments and reagents

All solvents in the experiments were treated with 5 Å molecular sieves. Melting points were determined using a WRS-1B microscopic and a melting point instrument (Shanghai YiCe Apparatus & Equipment Co. Ltd) without correction. NMR spectra were acquired on a Varian Mercury 600. ^1^H NMR and ^13^C NMR were calibrated using tetramethylsilane. Infrared spectra were measured on a Bruker Tensor 27 Infrared spectrometer. Mass spectra were obtained using a Bruker APEXIIFT-ICR mass spectrometer. Crystal diffraction analysis was performed using a AFC10/Saturn 724+ X-ray Single Crystal Diffractometer. Compounds 1a and 1b were prepared according to literature.^22,23^ Compounds 2a and 2d were prepared according to literature.^16,18^

### 5.2 Synthesis of target compounds 2b–2d

A general method was used to synthesize 2b–2d, and we provide the method for synthesis of 2b. In a 100 mL three neck flask, 1-formylferrocene (0.32 g, 1.5 mmol), 2-nitrophenanthraquinone (0.38 g, 1.5 mmol), ammonia acetate (0.58 g, 7.5 mmol) and 20 mL anhydrous methanol were mixed. The temperature was held at ∼65 °C and the solution left overnight. The mixture was cooled to room temperature and poured into a 30 mL mixture of ice and water. A brown-red solid was precipitated when the pH value was adjusted to 8. After filtration and washing with dichloromethane, the brown-red product 2b was obtained.

#### 2-Ferrocenyl-1*H*-2-nitrophenanthro[9,10-*d*]imidazole (2b)

580 mg brown-red solid, yield: 86.8%. Mp >270 °C; ^1^H NMR (DMSO-*d*_*6*_, 600 MHz): *δ* 13.24 (d, *J* = 85.5 Hz, 1H), 9.38 (dd, *J* = 135.2, 2.4 Hz, 1H), 9.09 (dd, *J* = 13.5, 9.2 Hz, 1H), 8.93 (dd, *J* = 23.7, 8.3 Hz, 1H), 8.57 (dd, *J* = 16.5, 7.9 Hz, 1H), 8.32 (ddd, *J* = 8.7, 4.8, 2.5 Hz, 1H), 7.85 (dt, *J* = 31.0, 7.4 Hz, 1H), 7.75–7.66 (m, 1H), 5.20 (d, *J* = 29.6 Hz, 2H), 4.58–4.51 (m, 2H), 4.17 (d, *J* = 2.3 Hz, 5H); ^13^C NMR (DMSO-*d*_*6*_, 151 MHz): *δ* 151.8, 145.7, 145.6, 138.2, 136.6, 131.4, 131.2, 129.0, 128.4, 128.0, 126.9, 126.4, 126.2, 126.0, 125.8, 125.7, 125.6, 125.4, 125.0, 123.5, 122.3, 122.2, 121.9, 118.0, 117.7, 117.3, 109.7, 74.4, 69.8, 69.5, 67.3, 67.2; HRMS (Thermo Q Exactive) calcd for C_25_H_17_FeN_3_O_2_ 448.0743, found 448.0730.

#### 2-(1′-Acetyl)ferroenyl-1*H*-phenanthro[9,10-*d*]imidazole (2c)

378 mg, brown solid, yield: 56.5%. Mp >236 °C; ^1^H NMR (DMSO-*d*_*6*_, 600 MHz): *δ* 13.02 (s, 1H), 8.87 (d, *J* = 8.3 Hz, 1H), 8.83 (d, *J* = 8.3 Hz, 1H), 8.54 (d, *J* = 7.9 Hz, 1H), 8.46 (d, *J* = 7.9 Hz, 1H), 7.78–7.73 (m, 1H), 7.73–7.68 (m, 1H), 7.66–7.59 (m, 2H), 5.25 (s, 1H), 4.75 (s, 1H), 4.56 (s, 1H), 4.52 (s, 1H), 2.11 (s, 3H); ^13^C NMR (DMSO-*d*_*6*_, 151 MHz): *δ* 201.2, 149.0, 137.3, 127.8, 127.6, 127.5, 127.4, 127.3, 125.4, 125.3, 124.5, 124.1, 122.7, 122.4, 122.3, 80.8, 77.5, 73.8, 73.7, 71.4, 71.0, 68.6, 27.7; HRMS (Thermo Q Exactive) calcd for C_27_H_20_FeN_2_O 445.0998, found 445.0997.

#### 2-(1′-Acetyl)ferrocenyl-1*H*-(2-itrophenanthro)[9,10-*d*]imidazole (2d)

490 mg, brown red solid, yield: 66.7%. Mp >194 °C; ^1^H NMR (DMSO-*d*_*6*_, 600 MHz): *δ* 13.29 (d, *J* = 84.7 Hz, 1H), 9.37 (d, *J* = 120.7 Hz, 1H), 9.11 (s, 1H), 8.94 (d, *J* = 24.3 Hz, 1H), 8.56 (d, *J* = 23.9 Hz, 1H), 8.34 (s, 1H), 7.86 (d, *J* = 31.7 Hz, 1H), 7.72 (s, 1H), 5.26 (d, *J* = 32.7 Hz, 2H), 4.78 (s, 2H), 4.57 (d, *J* = 34.2 Hz, 4H), 2.10 (d, *J* = 4.5 Hz, 3H); ^13^C NMR (DMSO-*d*_*6*_, 151 MHz): *δ* 200.8, 150.0, 149.9, 145.7, 145.6, 138.3, 136.5, 131.5, 131.4, 129.1, 128.5, 128.1, 127.1, 126.4, 126.4, 126.3, 126.0, 125.8, 125.7, 125.4, 125.0, 123.5, 122.2, 121.9, 118.2, 117.7, 117.2, 80.5, 76.4, 73.4, 71.3, 70.7, 68.4, 68.4, 27.3; HRMS (Thermo Q Exactive) calcd for C_27_H_19_FeN_3_O_3_ 490.0849, found 490.0845.

## Conflicts of interest

There are no conflicts to declare.

## Supplementary Material

RA-008-C7RA12700F-s001

RA-008-C7RA12700F-s002
