# Supplementary material for: The first colorimetric receptor for the B4O72− anion based on nitro substituted phenanthroimidazole ferrocene derivatives
Source: RSC Adv. 2018 Jan 19;8(7):3782–8. doi: 10.1039/c7ra12700f (PMC9077699; doi:10.1039/c7ra12700f)
Supplement: RA-008-C7RA12700F-s001 [file RA-008-C7RA12700F-s001.pdf]

Electronic Supplementary Material (ESI) for RSC Advances.
This journal is © The Royal Society of Chemistry 2018

Electronic Supplementary Information

The first colorimetric receptor for the $B_4O_7^{2-}$ anion based on nitro substituted phenanthroimidazole ferrocene derivatives

Pei Wu, Guo Wang, Lu Zhou, Jing Lu and Jianchun Wang*

Department of Chemistry, Capital Normal University, Beijing, 100048, PR China
*Corresponding Author, tel.: +86-010-68902974, e-mail: cnuwjc@cnu.edu.cn.

Table of Contents

1. **Benesi-Hildebrand plots**
2. **Crystal data for compound 2a.**
3. **^{1}H NMR and ^{13}C NMR spectra for 2b-2d.**
4. **HRMS (Thermo Q Exactive) spectra for 2b-2d.**

1. Benesi-Hildebrand plots

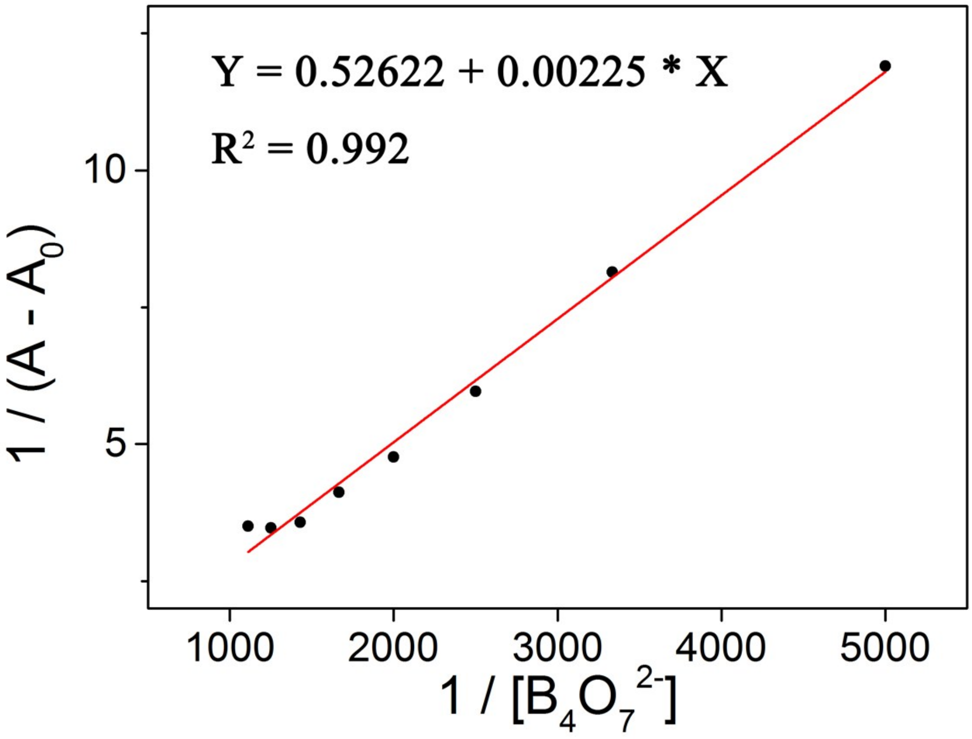

Figure S1. Benesi-Hildebrand analysis of **2b** at different $B_4O_7^{2-}$ concentrations.

2. Crystal data for compound 2a

Table S1 Crystal data and structural refinement for **2a**

Complex	**2a**
Formula	$C_{25}H_{18}FeN_2$
Crystal system	Orthorhombic
Space group	C222(1)
a (Å)	9.746(2)
b (Å)	19.123(4)
c (Å)	19.154(4)
α (°)	90.00
β (°)	90.00
γ (°)	90.00
V (Å^3)	3750.1(13)
Z	8
F(000)	1664
Reflections collected	5997
Independent reflections	22915
Goodness-of-fit on F^2	1.003
R(reflections)	0.0541(4640)
wR2(reflections)	0.1207(5921)

Table S2 Selected bond length (Å) and bond angles (˚) in **2a**

bond(Å)	angle(˚)	bond(Å)	angle(˚)	bond(Å)	angle(˚)
Fe(1)—C(6)	2.043(3)	Fe(1)—C(3)	2.044(3)	Fe(1)—C(9)	2.045(3)
Fe(1)—C(9)	2.046(3)	Fe(1)—C(4)	2.047(3)	Fe(1)—C(7)	2.050(3)
Fe(1)—C(1)	2.055(3)	Fe(1)—C(8)	2.056 (3)	Fe (1)—C(5)	2.056(3)
Fe(1)—C(10)	2.059(3)	N(1)—C(11)	1.353(4)	N(1)—C(12)	1.393(4)
N(2)—C(11)	1.356(4)	N(2)—C(25)	1.398(4)	C(1)—C(2)	1.403(5)
N(1)—C(13)	1.311(8)	N(1)—C(14)	1.394(4)	C(3)—C(4)	1.416(5)
C(1)—C(5)	1.411(5)	C(2)—C(3)	1.408(5)	C(3)—C(4)	1.416(5)
C(4)—C(5)	1.406(5)	C(6)—C(7)	1.419(4)	C(6)—C(10)	1.432(4)
C(7)—C(8)	1.448(4)	C(7)—C(11)	1.460(4)	C(8)—C(9)	1.420(4)
C(9)—C(10)	1.414(5)	C(12)—C(25)	1.374(4)	C(12)—C(13)	1.441(4)
C(13)—C(14)	1.415(4)	C(13)—C(18)	1.419(4)	C(14)—C(15)	1.377(5)
C(15)—C(16)	1.393(5)	C(16)—C(17)	1.361(5)	C(17)—C(18)	1.410(4)
C(18)—C(19)	1.480(5)	C(19)—C(20)	1.415(4)	C(19)—C(24)	1.417(4)
C(20)—C(21)	1.372(5)	C(21)—C(22)	1.405(5)	C(22)—C(23)	1.373(5)
C(23)—C(24)	1.422(4)	C(24)—C(25)	1.429(4)	C(6)—Fe(1)—C(3)	156.85(14)
C(6)—Fe(1)—C(9)	68.17(12)	C(3)—Fe(1)—C(9)	125.26(14)	C(6)—Fe(1)—C(4)	160.93(13)
C(3)—Fe(1)—C(4)	40.52(15)	C(9)—Fe(1)—C(4)	107.99(13)	C(6)—Fe(1)—C(7)	40.60(11)
C(3)—Fe(1)—C(7)	121.88(13)	C(9)—Fe(1)—C(7)	68.38(11)	C(4)—Fe(1)—C(7)	157.18(13)
C(6)—Fe(1)—C(2)	121.62(14)	C(3)—Fe(1)—C(2)	40.23(15)	C(9)—Fe(1)—C(2)	161.95(14)
C(4)—Fe(1)—C(2)	67.83(14)	C(7)—Fe(1)—C(2)	108.24(13)	C(6)—Fe(1)—C(1)	108.15(13)
C(3)—Fe(1)—C(1)	67.33(14)	C(9)—Fe(1)—C(1)	156.45(13)	C(4)—Fe(1)—C(1)	67.45(13)
C(7)—Fe(1)—C(1)	124.96(12)	C(2)—Fe(1)—C(1)	39.97(14)	C(6)—Fe(1)—C(8)	68.89(12)
C(3)—Fe(1)—C(8)	107.97(14)	C(9)—Fe(1)—C(8)	40.52(12)	C(4)—Fe(1)—C(8)	121.18(13)
C(7)—Fe(1)—C(8)	41.32(11)	C(2)—Fe(1)—C(8)	125.26(14)	C(1)—Fe(1)—C(8)	161.96(13)
C(6)—Fe(1)—C(5)	124.63(13)	C(3)—Fe(1)—C(5)	67.60(14)	C(9)—Fe(1)—C(5)	121.40(12)
C(4)—Fe(1)—C(5)	40.08(14)	C(7)—Fe(1)—C(5)	161.25(12)	C(2)—Fe(1)—C(5)	67.55(14)
C(1)—Fe(1)—C(5)	40.15(13)	C(8)—Fe(1)—C(5)	156.11(13)	C(6)—Fe(1)—C(10)	40.87(12)
C(3)—Fe(1)—C(10)	161.21(14)	C(9)—Fe(1)—C(10)	169.8(3)	C(4)—Fe(1)—C(10)	124.17(13)
C(7)—Fe(1)—C(10)	68.59(11)	C(2)—Fe(1)—C(10)	156.66(14)	C(1)—Fe(1)—C(10)	121.58(14)
C(8)—Fe(1)—C(10)	68.61(13)	C(5)—Fe(1)—C(10)	107.60(13)	C(11)—N(1)—C(12)	104.6(2)
C(1)—N(2)—C(25)	104.8(2)	C(2)—C(1)—C(5)	108.4(3)	C(2)—C(1)—Fe(1)	69.85(18)
C(5)—C(1)—Fe(1)	69.96(18)	C(1)—C(2)—C(3)	107.9(3)	C(1)—C(2)—Fe(1)	70.18(18)
C(3)—C(2)—Fe(1)	69.64(18)	C(2)—C(3)—C(4)	108.0(3)	C(2)—C(3)—Fe(1)	70.13(19)
C(4)—C(3)—Fe(1)	69.82(18)	C(5)—C(4)—C(3)	107.8(3)	C(5)—C(4)—Fe(1)	70.32(18)
C(3)—C(4)—Fe(1)	69.66(19)	C(4)—C(5)—C(1)	107.9(3)	C(4)—C(5)—Fe(1)	69.59(18)
C(1)—C(5)—Fe(1)	69.89(18)	C(7)—C(6)—C(10)	108.5(3)	C(7)—C(6)—Fe(1)	69.86(16)
C(10)—C(6)—Fe(1)	70.15(16)	C(6)—C(7)—C(8)	107.9(3)	C(6)—C(7)—C(11)	126.9(3)
C(8)—C(7)—C(11)	125.0(3)	C(6)—C(7)—Fe(1)	69.54(16)	C(8)—C(7)—Fe(1)	69.65(15)
C(11)—C(7)—Fe(1)	123.22(19)	C(9)—C(8)—C(7)	106.6(3)	C(9)—C(8)—Fe(1)	69.34(16)
C(7)—C(8)—Fe(1)	69.03(15)	C(10)—C(9)—C(8)	109.8(3)	C(10)—C(9)—Fe(1)	70.35(16)
C(8)—C(9)—Fe(1)	70.14(16)	C(9)—C(10)—C(6)	107.2(3)	C(9)—C(10)—Fe(1)	69.33(17)

C(6)—C(10)—Fe(1)	68.98(16)	N(1)—C(11)—N(2)	113.4(3)	N(1)—C(11)—C(7)	124.6(3)
N(2)—C(11)—C(7)	122.0(3)	C(25)—C(12)—N(1)	109.1(3)	C(25)—C(12)—C(3)	121.9(3)
N(1)—C(12)—C(13)	129.0(3)	C(14)—C(13)—C(18)	120.0(3)	C(14)—C(13)—C(12)	122.6(3)
C(18)—C(13)—C(12)	117.4(3)	C(15)—C(14)—C(13)	120.0(3)	C(14)—C(15)—C(16)	68.98(16)
C(14)—C(13)—C(12)	122.0(3)	C(14)—C(13)—C(12)	122.0(3)	C(14)—C(13)—C(12)	120.0(3)
C(17)—C(16)—C(15)	120.9(3)	C(16)—C(17)—C(18)	121.5(3)	C(17)—C(18)—C(13)	117.6(3)
C(17)—C(18)—C(19)	122.1(3)	C(13)—C(18)—C(19)	120.2(3)	C(20)—C(19)—C(24)	118.0(3)
C(20)—C(19)—C(18)	121.9(3)	C(24)—C(19)—C(18)	120.1(3)	C(21)—C(20)—C(19)	121.2(3)
C(20)—C(21)—C(22)	120.5(3)	C(23)—C(22)—C(21)	120.2(3)	C(22)—C(23)—C(24)	120.0(3)
C(19)—C(24)—C(23)	120.0(3)	C(19)—C(24)—C(25)	117.6(3)	C(23)—C(24)—C(25)	122.4(3)
C(12)—C(25)—N(2)	108.1(2)	C(12)—C(25)—C(24)	122.7(3)	N(2)—C(25)—C(24)	129.2(3)

3. ^{1}H NMR and ^{13}C NMR spectra for 2b-2d

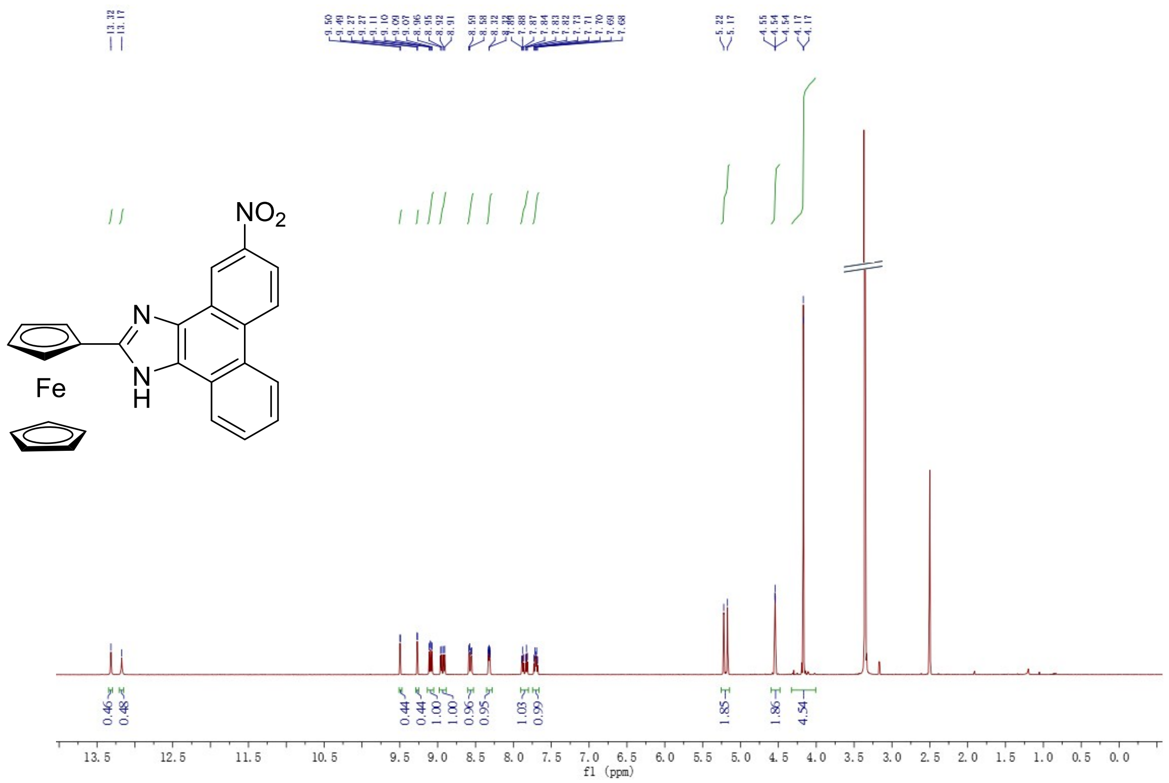

Fig. S2 ^{1}H NMR spectrum of compound **2b**.

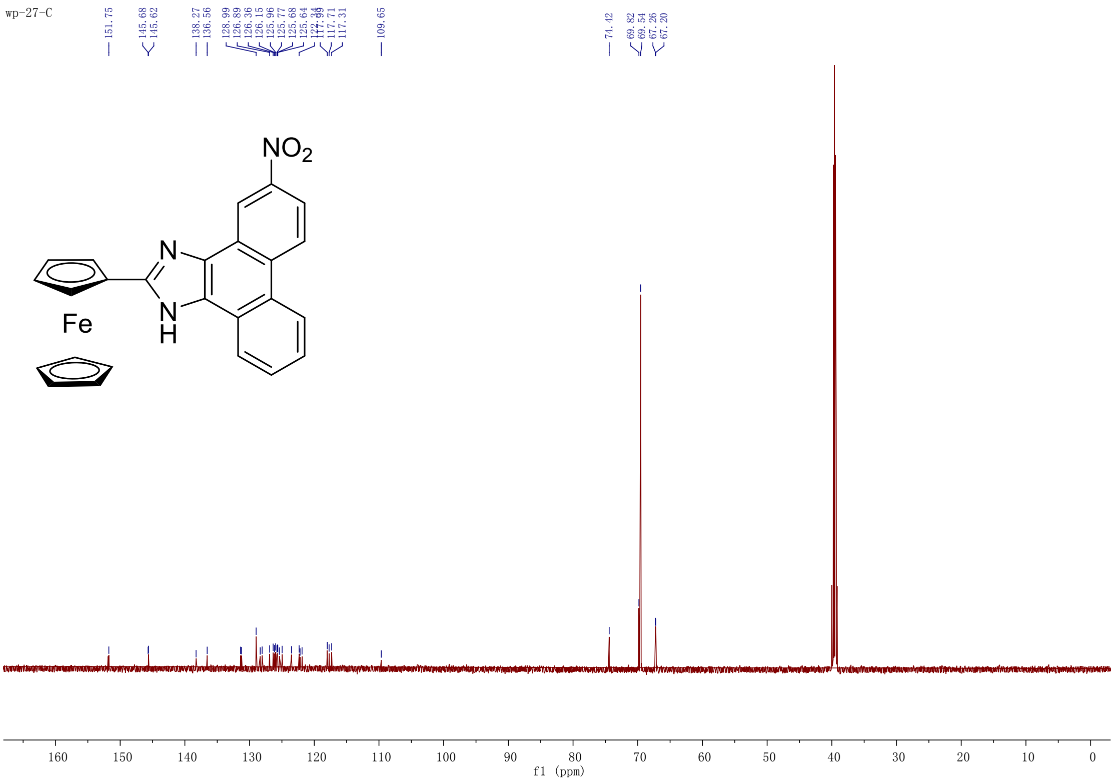

Fig. S3 ^{13}C NMR spectrum of compound **2b**.

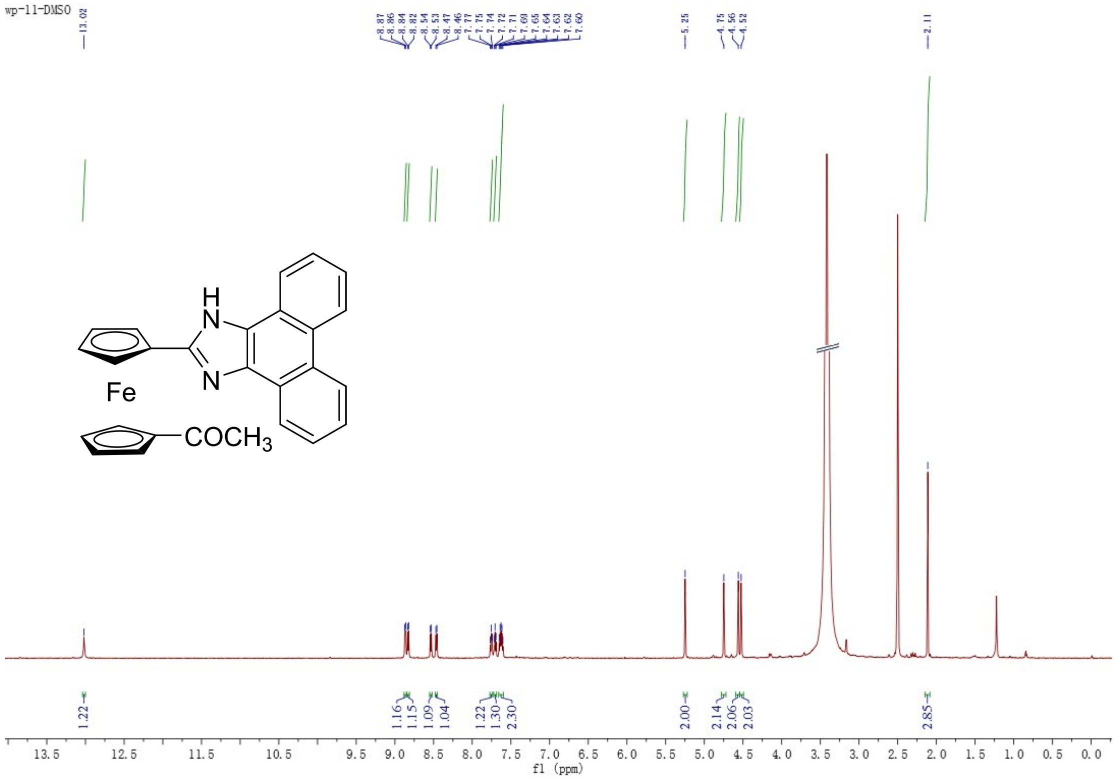

Fig. S4 ^{1}H NMR spectrum of compound **2c**.

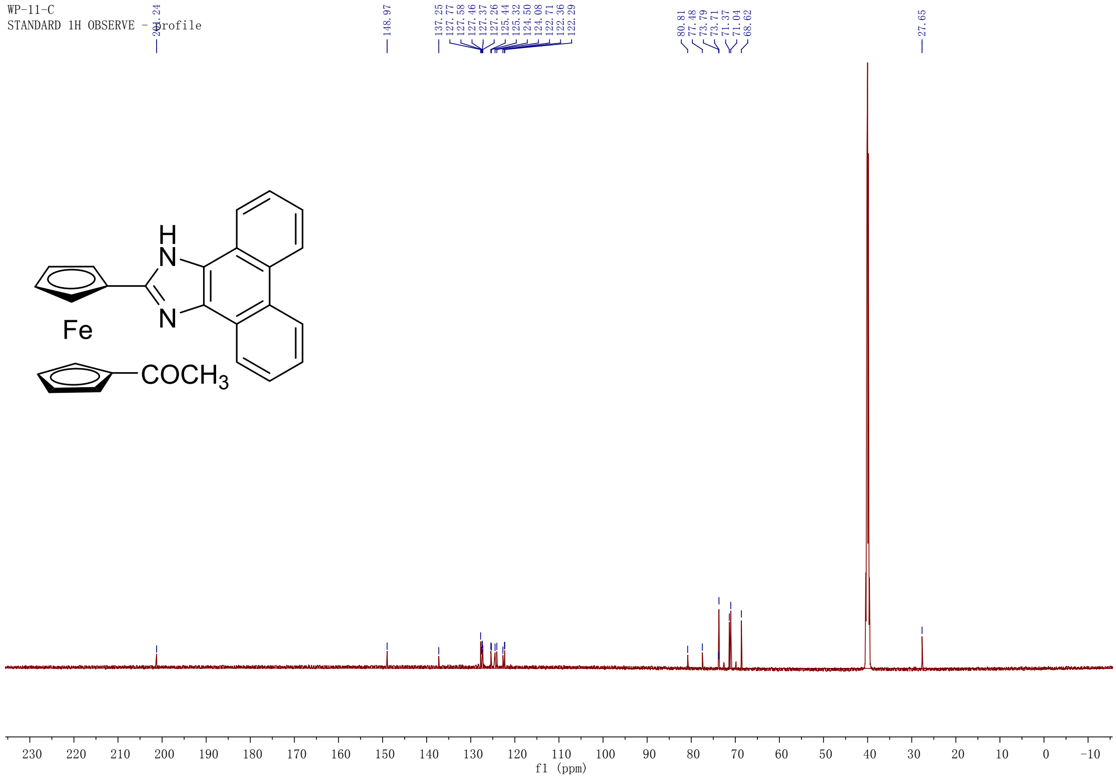

Fig. S5 ^{13}C NMR spectrum of compound **2c**.

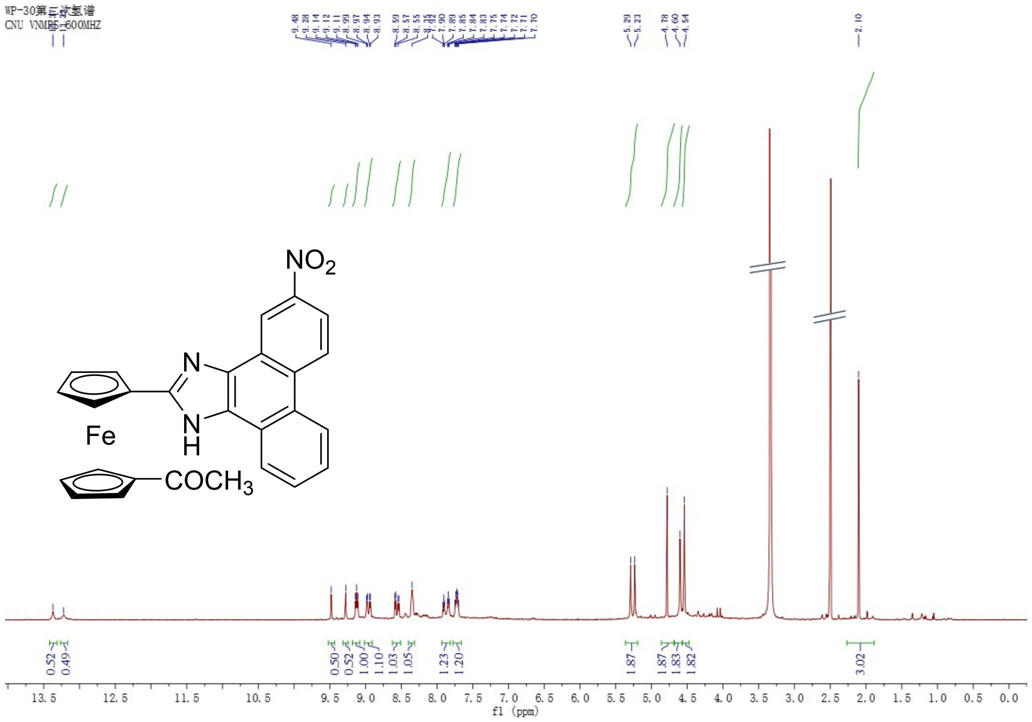

Fig. S6 ^{1}H NMR spectrum of compound **2d**.

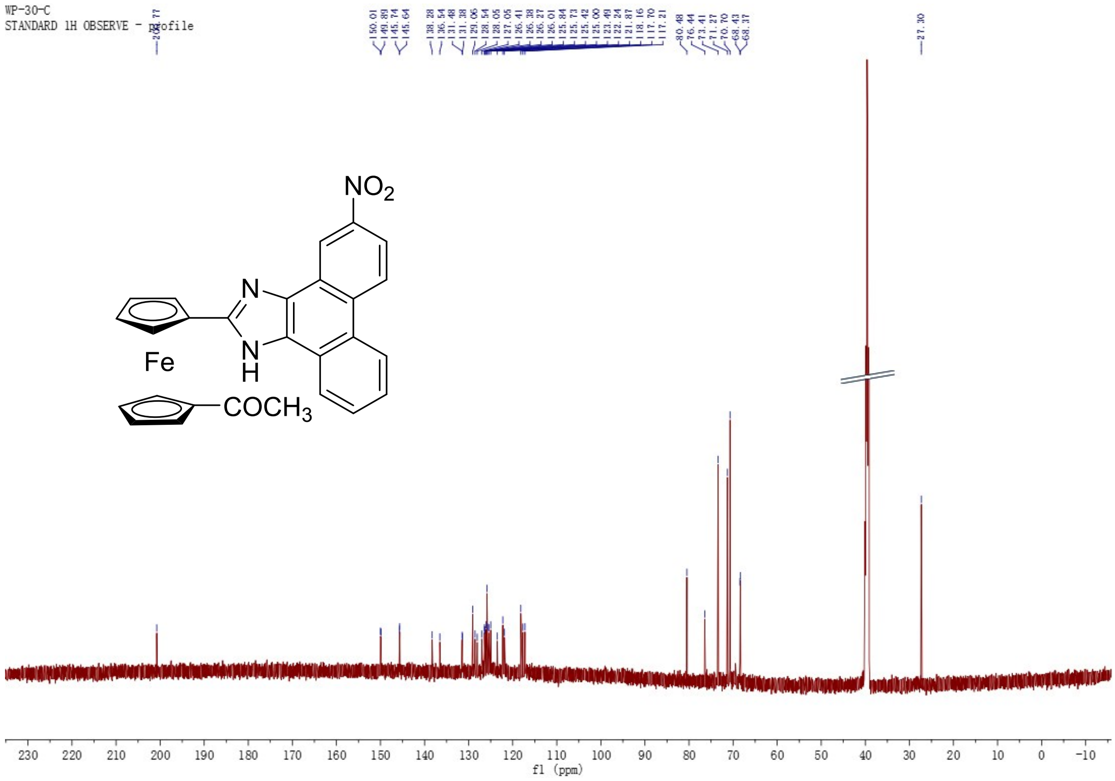

Fig. S7 ^{13}C NMR spectrum of compound **2d**.

4. HRMS (Thermo Q Exactive) spectra for 2b-2d.

HIGH RESOLUTION MASS SPECTROMETRY REPORT

Sample No.	Formula (M)	Ion Formula	Measured m/z	Calc m/z	Diff. (ppm)
**2b**	$C_{25}H_{17}FeN_3O_2$	$[M+H]^+$	448.0730	448.0743	2.90

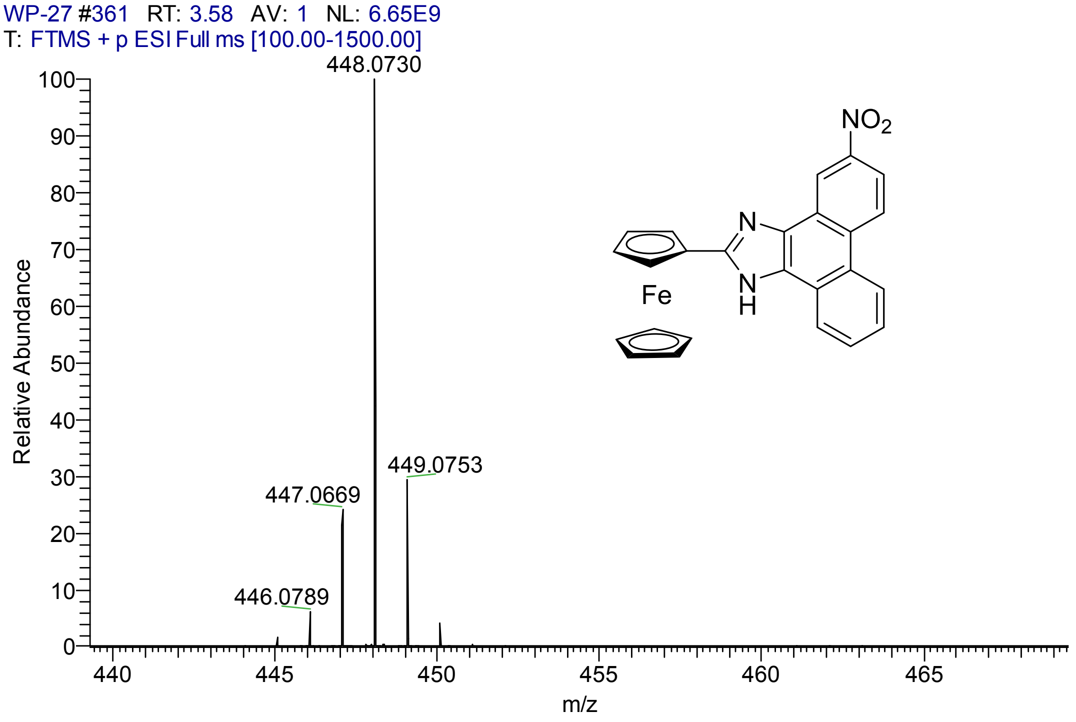

Fig. S8 HRMS data of compound **2b**.HIGH RESOLUTION MASS SPECTROMETRY REPORT

Sample No.	Formula (M)	Ion Formula	Measured m/z	Calc m/z	Diff. (ppm)
**2c**	$C_{25}H_{18}FeN_2$	$[M+H]^+$	445.0997	445.0998	2.25

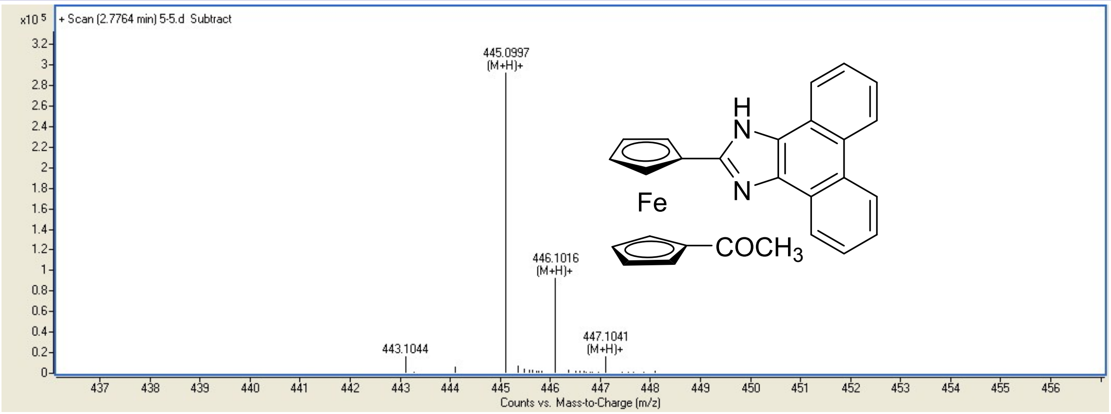

Fig. S9 HRMS results of compound **2c**.

HIGH RESOLUTION MASS SPECTROMETRY REPORT

Sample No.	Formula (M)	Ion Formula	Measured m/z	Calc m/z	Diff. (ppm)
**2d**	$C_{27}H_{19}FeN_3O_3$	$[M+H]^+$	490.0845	490.0849	0.82

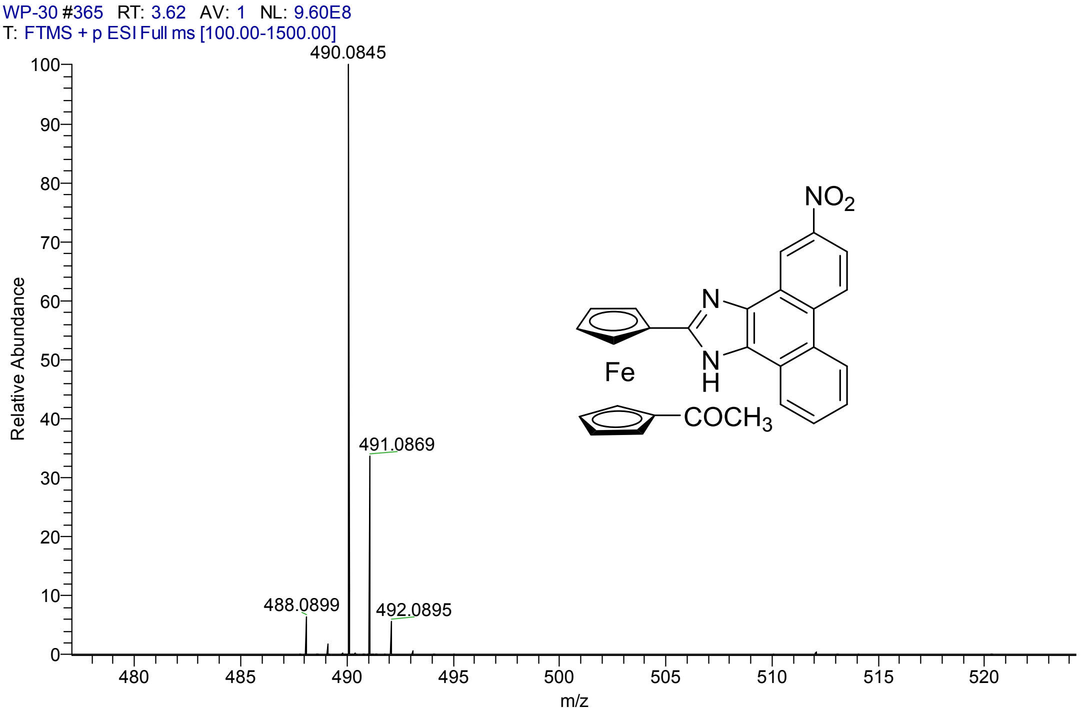

Fig. S10

HRMS results of compound **2d**.
